# Structural Characterization, Constipation-Relieving, and Hypolipidemic Activity of Polysaccharides from Fresh and Processed *Dendrobium officinale*

**DOI:** 10.3390/foods15040727

**Published:** 2026-02-15

**Authors:** Tingting Ding, Qingquan Ma, Xin Xu, Caiyue Chen, Ya Song, Xiang Zou, Shuqi Gao, Tingting Zhang, Fengzhong Wang, Jing Sun, Bei Fan

**Affiliations:** 1 Institute of Food Science and Technology, Chinese Academy of Agricultural Sciences, Laboratory of Quality and Safety Risk Assessment on Agro-Products Processing, Key Laboratory of Agro-Products Quality and Safety Control in Storage and Transport Process, Ministry of Agriculture and Rural Affairs, Beijing 100193, Chinawangfengzhong@caas.cn (F.W.); 2 School of Laboratory Medicine, Chengdu Medical College, Chengdu 610500, China; 3 Pharmaceutical Engineering Technology Research Center, Harbin University of Commerce, Harbin 150028, China; 4 National Center of Technology Innovation for Comprehensive Utilization of Saline-Alkali Land, Dongying 257000, China

**Keywords:** *Dendrobium officinale*, polysaccharides, purification, structural characterization, constipation relieving activity, hypolipidemic activity

## Abstract

*Dendrobium officinale* (DO) is a traditional medicinal and edible plant whose polysaccharides help modulate gastrointestinal and metabolic functions. Fresh DO is commonly processed into “Fengdou” to prolong shelf life, but the effects of this processing on polysaccharide structure and bioactivity remain unclear. In this study, polysaccharides from fresh DO (FDOP) and Fengdou (DDOP) were isolated, purified, and comparatively characterized. Based on structural analyses, FDOP and DDOP have similar functional groups and O-acetylated pyranosyl structures in both polysaccharides, which are identified as mannose–glucose heteropolysaccharides. However, FDOP was characterized by a higher mannose-to-glucose ratio (79.77:19.57) and molecular weight (187.1 kDa), as well as a more structurally diversified →4-linked backbone. In contrast, DDOP contained more glucose (68.74:30.94) and exhibited a lower molecular weight (125.1 kDa) and simplified backbone. In zebrafish models, both polysaccharides were found to alleviate loperamide-induced constipation and reduce lipid accumulation. DDOP showed stronger constipation-relieving activity, whereas FDOP exerted more pronounced hypolipidaemic effects, which can be ascribed to the higher molecular weight, mannose enrichment, and more complex backbone structure. These findings provide a structural basis and theoretical support for developing DO-derived polysaccharides as functional food ingredients targeting constipation and dyslipidaemia.

## 1. Introduction

*Dendrobium officinale* (DO) is a highly valued medicinal and edible plant, esteemed in traditional Chinese practice through its long-standing use [1]. Due to its perishable nature in fresh form, bioactive components in DO are susceptible to degradation or loss during storage and transport. To overcome this limitation, DO is commonly processed into “Fengdou” to enable long-term preservation and commercial distribution [2,3]. DO polysaccharides demonstrate diverse biological effects such as antioxidant, anti-aging, constipation-relieving, and hypolipidemic activities, which are among the key bioactive components of this plant. Their low toxicity and excellent biocompatibility further make them more suitable than many synthetic drugs for long-term dietary intervention [4,5]. It is worth noting that thermal treatment and dehydration steps during processing may alter the composition and molecular conformation of polysaccharides, thereby significantly affecting their physicochemical properties and bioactivities [6,7,8]. However, systematic comparisons of the processing-induced alterations in the structural features and biological activities of DO polysaccharides remain scarce. Clarifying such changes is essential for understanding the structure–activity relationships of the bioactive components and for guiding the rational application of DO.

According to traditional Chinese medicine, DO is associated with the stomach and kidney meridians and has been historically used to “invigorate the stomach, promote fluid production, nourish yin, and clear heat” [9]. Modern pharmacological studies further indicate that DO has potential benefits in improving gastrointestinal function and regulating glucose and lipid metabolism [10,11,12].

Against this background, our study focuses on the effects of DO polysaccharides in relieving constipation and modulating blood lipids. Constipation and dyslipidaemia are increasingly common health issues and are pathophysiologically interrelated as intestinal motility and microbiota homeostasis influence lipid digestion and metabolism, while dyslipidaemia can, in turn, exacerbate intestinal dysfunction [13]. Therefore, identifying natural components capable of simultaneously improving intestinal motility and lipid metabolism holds significant clinical value.

Current clinical management of constipation agents such as polyethylene glycol, domperidone, and lactulose. Although these can alleviate symptoms, they produce side effects, including unpleasant taste, arrhythmia, and diarrhea, which limit their long-term safety and patient compliance [14]. Similarly, in the management of hyperlipidemia, although statins exert beneficial effects on lowering blood lipids, they may carry the risk of hepatotoxicity, elevated blood glucose, and gastrointestinal discomfort [15]. Meanwhile, hyperlipidemia is a major risk factor for cardiovascular morbidity. The rising incidence, with onset at increasingly younger ages [16,17], underscores the need for safer, long-term interventions.

Given these limitations, there is a growing need to identify natural products with higher safety profiles that possess both constipation-relieving and lipid-lowering activities, providing new candidates for functional foods and adjunctive therapies.

Zebrafish share up to 70% genome similarity with humans, and their intestinal tissue architecture, epithelial cell lineages, and peristaltic function are highly comparable to those of mammals, making them well-suited for investigating intestinal motility and related mechanisms [18,19]. In addition, key regulators of lipid metabolism, such as SREBF1, PPARα/γ, and ApoB, are conserved in zebrafish. Their physiological responses to high-fat or high-cholesterol diets closely resemble those observed in mammals, allowing the establishment of stable in vivo models of hyperlipidemia, hepatic steatosis, and systemic lipid accumulation. The optical transparency of zebrafish embryos and larvae further allows real-time imaging of lipid deposition and metabolic dynamics using lipophilic fluorescent probes or specific stains, providing an attractive high-throughput platform for screening natural compounds with lipid-modulating and gut-protective activities [20,21].

Taken together, this study was designed to isolate and purify polysaccharide fractions from fresh DO (FDOP) and its processed product, Fengdou (DDOP), and systematically compare their structural characteristics. Using zebrafish models as an in vivo model, we further evaluated the dual activities of DDOP and FDOP in alleviating constipation and regulating blood lipids. These findings are expected to serve as a rationale for developing DO-derived dietary polysaccharides with dual regulatory functions on intestinal and metabolic health.

## 2. Materials and Methods

### 2.1. Materials and Chemicals

Fresh stems of DO and the processed product Fengdou were obtained from Dendrobium Research Institute (Longling County, China). Processing was conducted in accordance with local standard procedures.

Chemical reagents, including Anhydrous ethanol, sodium hydroxide, hydrochloric acid, concentrated sulfuric acid, and sodium chloride, were all purchased from China National Pharmaceutical Group Chemical Reagent Co., Ltd. (Beijing, China). Trifluoroacetic acid, 4-nitrophenyllauric acid ester, pancreatic lipase, orlistat, and P-nitrophenyl butyrate (PNPB) were all purchased from Shanghai McLean Biochemical Technology Co., Ltd. (Shanghai, China). Anhydrous glucose, D-galacturonic acid, meta hydroxybiphenyl, and monosaccharides fucose (Fuc), rhamnose (Rha), arabinose (Ara), galactose (Gal), glucose (Glc), mannose (Man), gluconic acid (GlcA), and galacturonic acid (GalA) were all purchased from Solarbio Technology Co., Ltd. (Beijing, China). Formaldehyde, phenol, Oil Red O dye, Nile Red dye, Tricaine, and 3% methylcellulose were all purchased from Guangda Hengyi Technology Co., Ltd. (Beijing, China). Cellulose-DEAE-52 cellulose column (26 mm × 50 cm) and Sephadex-G-150 column (26 mm × 50 cm), Loperamide hydrochloride, domperidone, egg yolk powder, atorvastatin, and PBS buffer were all purchased from Yuanye Biotechnology Co., Ltd. (Shanghai, China). All other chemicals are analytical grade or chromatographic grade.

### 2.2. Extraction and Purification of Dendrobium officinale Polysaccharides

#### 2.2.1. Polysaccharide Extraction from *Dendrobium officinale*

DO polysaccharides were extracted with modifications following the protocol of Zhou et al. [22]. Fresh stems and Fengdou samples of equivalent dry weight were weighed and soaked in ultrapure water for 30 min at a solid-to-liquid ratio of 1:20 (*w*/*v*). The mixtures were then incubated in hot water at 80 °C for 2 h. After extraction, the two filtrates were combined and concentrated to one-quarter of their original volume. The polysaccharides were precipitated by adding anhydrous ethanol slowly to the concentrate with constant stirring to a final concentration of 80% (*v*/*v*). The resulting precipitates were repeatedly washed with 80% ethanol and dried at room temperature. The dried product was then re-dissolved, mixed with 4% activated carbon, and decolorized at 40 °C for 1 h. After centrifugation, the supernatant was mixed with reagent (chloroform:n-butanol = 4:1) in a 1:4 (*v*/*v*) ratio to remove proteins. The deproteinized supernatant was then subjected to rotary evaporation to remove residual organic solvents and concentrated appropriately. The resulting concentrate was dialyzed against distilled water for 3 days with water changes every 4 h. Finally, the solution was freeze-dried to obtain two groups of crude DO polysaccharides.

#### 2.2.2. Purification of DO Polysaccharides

This purification step was carried out based on the method of Hui et al., with slight modifications [23]. 500 mg crude polysaccharide was initially fractionated by anion-exchange chromatography on a DEAE-52 cellulose column (26 mm × 50 cm) using a linear gradient of 0–0.5 M NaCl. All collected eluates were subjected to ultrasonic treatment to ensure homogeneity. Further purification was performed by gel filtration on a Sephadex G-150 column (26 mm × 50 cm). The carbohydrate content in the eluted fractions was determined by the phenol–sulfuric acid method according to the absorbance recorded at 490 nm. Based on the resulting elution profile, target fractions were pooled, concentrated, dialyzed, and finally lyophilized under vacuum for storage. The purified polysaccharides obtained from fresh and dried stems of *Dendrobium officinale* were designated as FDOP and DDOP, respectively.

### 2.3. Ultraviolet-Visible Spectroscopy (UV-Vis) Analysis

Accurately weigh 10.0 mg of FDOP and DDOP, respectively, and dissolve them to prepare polysaccharide solutions at a concentration of 1 mg/mL. The solutions are then placed in quartz cuvettes and scanned across a wavelength range of 190–500 nm using a UV spectrophotometer (Shimadzu, Kyoto, Japan) with ultrapure water as the background reference [24].

### 2.4. Molecular Weight Determination

The molecular weight distribution of the two polysaccharides was determined using high-performance liquid chromatography coupled with multi-angle light scattering detection (HPLC-MALS) [25], utilizing a Waters HPLC system (G4288C, Waters Corporation, Milford, MA, USA) equipped with an 18-angle light scattering detector (HPSEC-MALLS-RI, Wyatt Technology Corporation, Santa Barbara, CA, USA) and a Shodex 806 column (Shodex, Tokyo, Japan). The mobile phase was a 0.1 M sodium chloride solution, delivered at a flow rate of 0.5 mL/min with the column temperature maintained at 35 °C. A series of dextran standards (2 mg/mL in ultrapure water) and the polysaccharide samples (10 mg in 5 mL of 0.1 M NaCl) were filtered over a 0.45 μm aqueous membrane, and 100 μL of each was injected for HPLC-MALS analysis.

### 2.5. FT-IR Spectroscopy

The polysaccharide samples were finely ground with anhydrous potassium bromide and pressed into transparent pellets. FT-IR spectra were recorded (TENSOR 27, Bruker Corporation, Denver, CO, USA) across the range of 4000 cm^−1^ to 400 cm^−1^ [26].

### 2.6. Scanning Electron Microscopy (SEM)

FDOP and DDOP samples were horizontally placed on the sample stage with uniform thickness and securely pressed into position. After gold coating, the samples were transferred to a Hitachi SU8010 instrument (Hitachi, Tokyo, Japan) for observation at an accelerating voltage of 10.0 kV. The microstructural morphology of the samples was examined at a magnification of 500×.

### 2.7. Monosaccharide Analysis

Monosaccharide composition was detected by ion chromatography (IC) [27]. Briefly, 10 mg of FDOP and DDOP were placed into a hydrolysis tube. Then 4 mL of 4 mol/L trifluoroacetic acid (TFA) was added, and the tube was flushed with nitrogen for 1 min to exhaust the air from the tube. The polysaccharides were hydrolyzed at 120 °C for 4 h after tightening the screw cap. Upon cooling, the hydrolysate was evaporated to dryness under a stream of nitrogen. Methanol was then added to remove residual TFA, followed by the addition of ultrapure water to achieve a 20-fold dilution, and then filtered over a 0.2 µm membrane. Chromatographic separation was performed using a CarboPacTMPA20 column (3 × 150 mm) (Dionex 060142, Dionex, Sunnyvale, CA, USA). A pulsed amperometric detector (HPAEC-PAD, Waters Corporation, Milford, MA, USA) with a gold electrode was employed. The mobile phase consisted of ultrapure water, 250 mM NaOH, and 1 M NaAc, delivered at a flow rate of 0.5 mL/min. The column temperature was maintained at 35 °C, and the injection volume was 10 μL. The conditions of the mobile phase are detailed in Table S1.

### 2.8. Methylation Assay

The methylation analysis was conducted in accordance with the method from Zhang et al. [28]. After post-treatment, the samples were hydrolyzed with 2 M TFA (120 °C, 2 h). The hydrolysates were subsequently reduced with NaBD_4_ and acetylated with acetic anhydride to obtain partially methylated alditol acetates (PMAAs). The acetylated derivatives were subjected to GC–MS analysis on an HP-5 MS capillary column (30 m × 0.25 mm, 0.25 μm; Agilent, Santa Clara, CA, USA) with the temperature programmed from 120 °C (2 min hold) to 280 °C at 4 °C/min, followed by a 5 min isothermal period, using helium as the carrier gas. Data processing was performed using the MassHunter software (version B.07.00, Agilent Technologies, Santa Clara, CA, USA). Compounds were identified by comparing their mass spectra and relative retention times with the reference spectra of authentic PMAA standards in the National Institute of Standards and Technology (NIST) mass spectral library. The molar ratios of the constituent sugar residues were determined from their respective peak areas.

### 2.9. NMR Spectroscopy

50 mg of FDOP and DDOP were dissolved completely in 500 µL of D_2_O, respectively. The ^1^H NMR, ^13^C NMR, COSY, HSQC, and HMBC spectra were recorded using a Bruker spectrometer (Fourier 80, Bruker Corporation, Billerica, MA, USA) (700 MHz).

### 2.10. Animal Experimental Design

For this study, zebrafish (wild-type AB strain) were housed in a laboratory recirculating aquaculture system. All fish were obtained from the China Zebrafish Resource Center. All experimental procedures were approved by the Ethics Committee of the Institute of Agro-Products Processing, Chinese Academy of Agricultural Sciences (Approval No. 230320). The environmental temperature was maintained at 28–30 °C, with feeding occurring twice daily (in the morning and evening) under a 14 h light and 10 h dark cycle. Zebrafish were bred through natural pairing, and tank conditions were regularly maintained to ensure optimal water quality and animal welfare.

### 2.11. Constipation-Relieving Activity Test

The zebrafish constipation model was established based on the method of Wang et al., with minor modifications [29]. Briefly, 5 days post-fertilization (dpf) larvae were incubated with 10 μg/L Nile Red for 16 h to fluorescently label intestinal lipids, followed by three rinses with embryo culture water. To induce constipation, larvae were exposed to 10 μg/mL loperamide hydrochloride for 24 h to inhibit intestinal motility. A control group was maintained in embryo culture water under the same conditions. After treatment, zebrafish were euthanized by immersion in 0.2% (*w*/*v*)tricaine methanesulfonate (MS-222) and immobilized in 3% (*w*/*v*) methylcellulose to preserve intestinal morphology during imaging. Intestinal fluorescence intensity was quantified using ImageJ software (Fiji-Win64, v2.16.0), and statistical analyses were performed with SPSS to verify successful model establishment.

Constipated larvae were randomly divided into eight groups (*n* = 30 per group): a control group, a model group, a domperidone group, and groups treated with FDOP or DDOP at different concentrations. Dose levels were selected based on a preliminary tolerance assessment, in which no mortality was observed at 500–2000 μg/mL, whereas 40% mortality occurred at 2500 μg/mL and 100% mortality at 4000 μg/mL. Therefore, 2000 μg/mL was considered the maximum tolerated concentration and used as the upper limit for dose setting. After 24 h of administration, larvae were rinsed three times with embryo culture water. Intestinal motility was evaluated indirectly by measuring the fluorescence intensity of Nile Red in the gut, with higher fluorescence indicating greater retention of intestinal contents and more severe constipation.

### 2.12. Hypolipidemic Activity Test

#### 2.12.1. In Vivo Hypolipidemic Activity in Zebrafish

To model diet-induced lipid accumulation, larvae (5 dpf) were maintained in a 0.2% egg yolk powder suspension for 48 h to induce a diet-induced lipid load. The solution was replaced daily [30]. After modelling, larvae were randomly divided into eight groups (*n* = 30 per group): control group, model group, atorvastatin group (0.4 µM), and five FDOP- or DDOP-treated groups at 12.5, 25, 50, 100, and 200 µg/mL. Larvae were incubated with the respective treatments for 24 h. At the end of treatment, the egg yolk solution was removed, the fish were rinsed three times with PBS, and euthanized by immersion in 0.2% tricaine methanesulfonate (MS-222). The larvae were then fixed overnight at 4 °C using 4% paraformaldehyde, dehydrated through a graded methanol series (0, 25, 50, 75, and 100%), stained with 1 µg/mL Oil Red O, and rehydrated with methanol in reverse order. After three additional washes with PBS, the stained larvae were embedded in 3% (*w*/*v*) methylcellulose and photographed under a microscope. The integrated optical density (IOD) of Oil Red O-stained lipids was quantified using Image-Pro Plus software, and statistical analysis was performed with SPSS to evaluate lipid accumulation in vivo.

#### 2.12.2. In Vitro Pancreatic Cholesterol Esterase (PCE) and Pancreatic Lipase (PL) Activity Test

1. Inhibition of PCE

The inhibitory activity of FDOP and DDOP against PCE was assessed using a protocol adapted from Long et al. [31]. Briefly, PCE and polysaccharide samples were separately dissolved in distilled water, with the latter serially diluted to target concentrations. The reaction system consisted of 1.0 mL of phosphate buffer (5.16 mmol/L, pH 7.0, containing 0.10 mol/L NaCl), 100 µL of enzyme solution, and 100 µL of polysaccharide sample. After a 5 min pre-incubation at 25 °C, the reaction was started by adding 50 µL of p-nitrophenyl butyrate (PNPB) substrate. Following a 30 min incubation at the same temperature, the absorbance of the released p-nitrophenol was recorded at 405 nm. The inhibition rate was calculated as follows:

Inhibition rate (%) = (A_1_ − A_2_ − (A_3_ − A_4_))/(A_1_ − A_2_) × 100% (1)

where A_1_, A_2_, A_3_, and A_4_ correspond to the absorbance of the control, blank background control, sample measurement, and sample background control, respectively. All assays were carried out with three independent replicates.

2. Inhibition of Pancreatic Lipase

The inhibitory effect of FDOP and DDOP on PL activity using a modified version of the method described by Chang et al. [32]. Reactions were carried out in 50 mmol/L phosphate buffer (pH 8.0) at 37 °C. Each 400 µL assay mixture was composed of 100 µL of polysaccharide sample, 100 µL of PL solution (10 mg/mL), and 200 µL of buffer containing the substrate. Specifically, after combining the sample, enzyme, and 100 µL of buffer for a 10 min pre-incubation, the reaction was commenced by adding 100 µL of 0.5 mmol/L 4-nitrophenyl laurate. Following a 20 min incubation, the enzymatic activity was quantified by measuring the increase in absorbance at 405 nm due to 4-nitrophenol release. The inhibition rate was calculated using the same formula as described above for PCE (Formula (1)), and all assays were performed in triplicate.

### 2.13. Statistical Analysis

Statistical analysis was performed using SPSS software (version 26.0). Multiple-group comparisons were conducted using one-way ANOVA followed by Tukey’s HSD post hoc test for multiple comparisons. A two-sided *p* < 0.05 was considered statistically significant. Data were then visualized and plotted with OriginPro 2021, GraphPad Prism 9.5, and MestReNova 15.0. Results are presented as the mean ± standard deviation (SD).

## 3. Results and Discussion

### 3.1. Isolation and Purification of Polysaccharides

**Figure 1:**
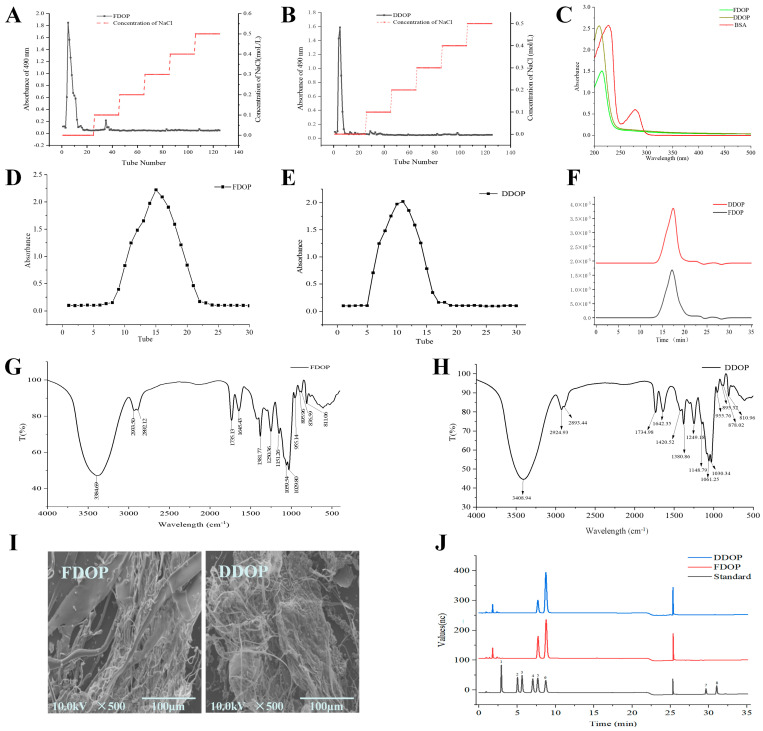
DEAE-52 elution curve of FDOP and DDOP (**A**,**B**); UV–vis spectrum of FDOP and DDOP (**C**); elution curve of Sephadex G150 of FDOP and DDOP (**D**,**E**); the molecular weight distribution of FDOP and DDOP (**F**); FT-IR spectrum of FDOP and DDOP (**G**,**H**); SEM images of FDOP and DDOP (500×) (**I**); IC spectra of monosaccharide standards, monosaccharide composition of FDOP and DDOP (**J**); 1—Fuc, 2—Rha, 3—Ara, 4—Gal, 5—Glc, 6—Man, 7—GalA, 8—GlcA.

The extraction yields of the crude polysaccharides were 18.74% for FDOP and 15.52% for DDOP. This difference in yield suggests that processing may affect polysaccharide recovery. Following dissolution in distilled water, the crude extracts were fractionated on a DEAE-52 cellulose column, yielding distinct elution profiles as shown in Figure 1A,B. To further enhance purity and homogeneity, the collected fractions were subjected to size-exclusion chromatography on a Sephadex G-150 column. The corresponding elution curves (Figure 1D,E) displayed symmetrical single peaks, confirming the successful isolation of homogeneous polysaccharide fractions. Final purity reached 89.4% ± 0.13% for FDOP and 84.5% ± 0.23% for DDOP, indicating high recovery and effective removal of non-polysaccharide impurities. FDOP and DDOP were found to contain 2.54% and 1.56% uronic acid, respectively, as determined by the m-hydroxybiphenyl method. The polysaccharide yield, purity, and uronic acid content were all consistently higher in FDOP than in DDOP. This suggests that FDOP may possess a more preserved or structurally distinct polysaccharide fraction. These compositional differences could contribute to variations in bioactivity, warranting further structural and functional characterization.

### 3.2. UV-Vis Analysis

Proteins and nucleic acids exhibit characteristic ultraviolet absorption at specific wavelengths, making UV spectroscopy a valuable method for detecting these impurities in polysaccharide samples. As shown in the full-wavelength UV scan (Figure 1C), neither FDOP nor DDOP exhibited absorption at these wavelengths, confirming the effective removal of such UV-absorbing impurities. This result validates the effectiveness of the purification steps applied and is consistent with reports on similar *Dendrobium* polysaccharides. It should be noted, however, that UV spectroscopy only detects impurities with characteristic chromophores. Therefore, sample homogeneity should be comprehensively assessed together with other analytical data, such as the single symmetric peak observed in gel chromatography.

### 3.3. Molecular Weight Analysis

The HPSEC-MALLS-RI analysis revealed that a single, symmetrical peak was observed in the refractive index (RI) chromatogram for both FDOP and DDOP, confirming the homogeneity of the purified polysaccharides (Figure 1F). Their weight-average molecular weights were determined to be 187.1 kDa for FDOP and 125.1 kDa for DDOP. This notable difference is likely attributable to the distinct processing histories of their source materials. FDOP was extracted from fresh *Dendrobium* stems, while DDOP was obtained from the processed product Fengdou. Traditional Fengdou processing involves steps such as hot-scalding, twisting, and drying. These procedures can hydrolyze and cleave glycosidic bonds in the polysaccharide chains, leading directly to a reduction in average molecular weight, as observed in DDOP [33]. The narrow molecular weight distribution further supports the high purity of both samples despite their size disparity.

### 3.4. FT-IR Analysis

The spectral profiles of FDOP and DDOP are shown in Figure 1G,H. Both polysaccharides displayed highly similar spectral profiles, confirming shared functional groups and structural motifs. A strong, broad band centered around 3400 cm^−1^, corresponding to O–H stretching vibrations, was dominant in both spectra. These features, along with other distinct absorption bands observed across the full scan range (4000–400 cm^−1^), are typical of polysaccharide structures. The presence of absorption peaks at 2933.50 cm^−1^ and 2892.12 cm^−1^ for FDOP and 2924.93 cm^−1^ and 2893.44 cm^−1^ for DDOP were asymmetric C–H stretching vibrations of sugars and may contain –CH_2_ or –CH_3_ [34]. In addition, signals indicative of O-acetylated pyranosyl structures were identified. These included bands around 1735 cm^−1^ (C=O stretch), 1380 cm^−1^ (C–H deformation), and 1250 cm^−1^ (C–O stretch), which are characteristic of the –O–COCH_3_ group. Additional bands near 1640 cm^−1^ and 1420 cm^−1^ were attributed to C=O and C–H stretching vibrations, respectively [35]. The spectral region between 1060 and 1030 cm^−1^ is typical of pyranose rings, while absorptions near 810 and 870 cm^−1^ are assigned to ring stretching and C_2_–H deformation vibrations specific to mannopyranose residues [36]. Furthermore, features near 1250 cm^−1^ were associated with O–H stretching and bending vibrations. Overall, FDOP and DDOP have highly similar spectra, indicating that both polysaccharides share comparable functional groups and contain O–acetylated pyranosyl structures.

### 3.5. SEM

The surface characteristics of FDOP and DDOP were examined under 500× magnification, revealing considerable differences between the two polysaccharides. As shown in Figure 1I, FDOP appeared as irregular flakes with a loose structure, consisting of small, smooth spherical particles and rod-like elements attached to fragmented flake assemblies. In contrast, DDOP exhibited a rougher surface with subtle bead-like textures and distinct corrugated folds, morphological features that may be attributed to processing-induced alterations.

### 3.6. Analysis of Monosaccharide Composition

The ion chromatography analysis results of the monosaccharide composition of the two polysaccharide fractions of DO are shown in Figure 1J. Both polysaccharides consisted predominantly of mannose and glucose, with other monosaccharides present at trace levels that could not be accurately quantified, which aligns with the findings of Zhao et al. [36]. The molar ratios were 79.8:19.6 and 68.8:30.9, respectively. This pattern, a reduced mannose content coupled with an elevated glucose level after processing, is consistent with the changes documented by Zhang et al. [37].

### 3.7. Methylation Analysis

**Figure 2:**
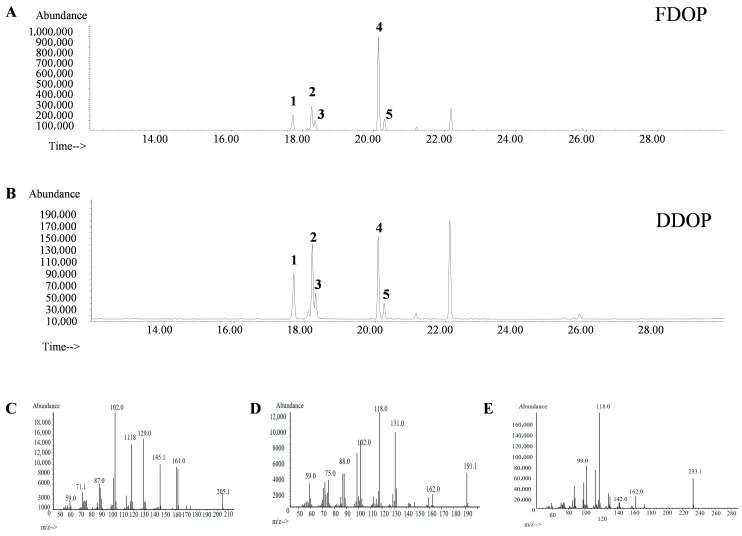
The methylation analysis of FDOP (**A**) and DDOP (**B**); Mass spectra of Peak 1 (**C**), Peak 2 and 3 (**D**), and Peak 4 and 5 (**E**).

To elucidate the glycosidic linkages and branching patterns, FDOP and DDOP were subjected to methylation analysis. As shown in Figure 2A,B, the predominant identified chain types were t-Glcp, 1,4-Glcp, 1,4-Manp, 1,4-Glcp (3-O-Ac), and 1,4-Manp (3-O-Ac), with their corresponding partial methylation patterns illustrated in Figure 2C–E [38]. PMAAs were characterized by their specific retention times and diagnostic fragment ions, with results compiled in Table 1. Their elution order followed the established trend where increased molecular weight and acetylation typically lead to longer retention [39]. Notably, the hydroxyl group at the 4-position remained unacetylated during the derivatization process, likely due to steric hindrance or reaction selectivity, resulting in the formation of 1,5-diacetyl-2,3,6-tri-O-methyl-4-hydroxyhexanol rather than the fully methylated derivative [40]. The glucomannan-type backbone of both polysaccharides was confirmed by methylation analysis, which revealed that 1,4-Glcp and 1,4-Manp residues were the dominant structural units, consistent with their monosaccharide compositions. This linkage fingerprint is consistent with recent methylation–GC–MS studies on DO polysaccharides, which commonly report 1,4-Glcp and 1,4-Manp as the predominant linkages, with minor →4,6-linked residues [41,42]. Notably, differences in the relative abundances of minor PMAAs among studies are expected. This variability arises because methylation analysis is sensitive to experimental conditions (e.g., methylation completeness, hydrolysis strength/duration, and potential changes in O-acetyl groups), as well as analytical configuration (e.g., column stationary phase, temperature program, and peak resolution/co-elution), which can influence PMAA separation and peak assignment. Therefore, the linkage ratios reported here are interpreted primarily as a comparative index between FDOP and DDOP under identical derivatization and GC–MS conditions. These methylation-derived data are further corroborated by 1D/2D NMR assignments to support the proposed structures.

### 3.8. NMR Spectral Analysis

Structural elucidation of the linkage patterns and glycosyl residues in FDOP and DDOP was carried out using NMR spectroscopy. In polysaccharides, analysis of the diagnostic anomeric region in NMR spectra allows for the identification of sugar residues [43].

**Figure 3:**
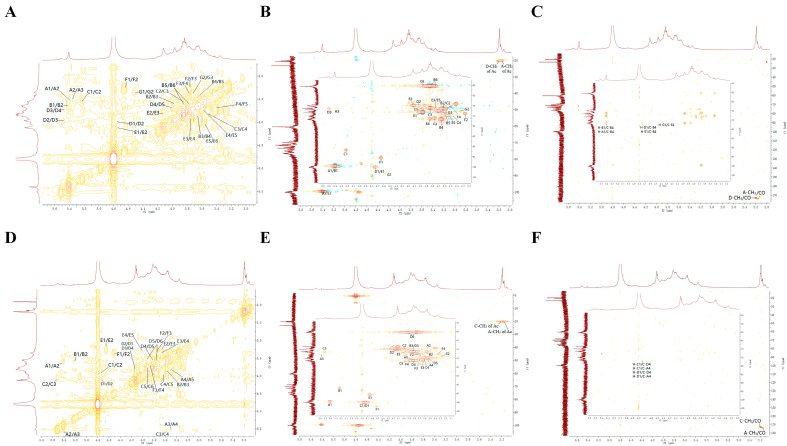
The COSY (**A**,**D**), HSQC (**B**,**E**), and HMBC (**C**,**F**) of FDOP and DDOP, respectively.

**Figure 4:**
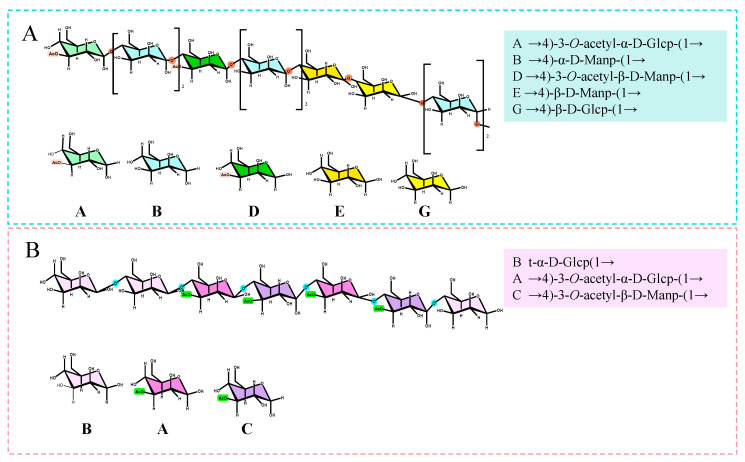
The prediction structure repetition unit spectra of FDOP (**A**) and DDOP (**B**), respectively.

As shown in the ^1^H NMR spectrum, FDOP exhibited multiple anomeric proton resonances, with chemical shifts recorded at δH 5.39, 5.39, 5.22, 4.73, 4.73, 4.65, and 4.51. Through analysis of the HSQC spectrum, these proton signals were correlated with anomeric carbon signals at δC 99.71, 99.71, 91.72, 100.08, 100.08, 96.95, and 102.44 in the ^13^C NMR spectrum, respectively. To facilitate the analysis and identification of sugar residues, the fragments were sequentially labeled as A through G. Structure elucidation was achieved by integrating data from monosaccharide composition, methylation analysis, and NMR spectroscopy. Specifically, the ^1^H and ^13^C chemical shifts for residues A–G were assigned using 1D (^1^H, ^13^C) and 2D (HSQC, HMBC, COSY) NMR experiments (Figure S1 and Figure 3A–C) [44,45]. This combined approach revealed that the FDOP backbone consists primarily of →4)-3-O-acetyl-α-D-Glcp-(1→, →4)-α-D-Manp-(1→, →4)-3-O-acetyl-β-D-Manp-(1→, →4)-β-D-Manp-(1→, and →4)-β-D-Glcp-(1→ residues (Figure 4A) [46,47,48]. The corresponding chemical shift data are summarized in Table S2. Notably, the structures shown in Figure 4 are proposed as repeating-unit models based on combined evidence and should not be interpreted as definitive full structures of the complete polysaccharide chains. The average chain size is instead approximated by the molecular weight determined by HPLC–MALS.

For DDOP, six anomeric proton signals were detected at δH 5.41, 5.23, 4.76, 4.76, 4.65, and 4.53, which correlated with carbon signals at δC 99.29, 91.73, 100.05, 100.05, 95.59, and 102.42 in the HSQC spectrum. Table S3 lists the detailed ^1^H and ^13^C chemical shifts for the glycosyl residues in FDOP/DDOP. These residues are designated by letters A to F, corresponding to a descending order of the ir anomeric proton δH values. The ^1^H and ^13^C signals of each residue (A–F) were further assigned using ^1^H NMR and ^13^C NMR (Figure S2), together with ^1^H–^1^H COSY, HSQC, and HMBC spectra (Figure 3D–F) [42,43]. Consequently, the backbone of DDOP was therefore inferred and is modeled as a repeating unit consisting mainly of →4)-3-O-acetyl-α-D-Glcp-(1→, →4)-3-O-acetyl-β-D-Manp-(1→ and →4)-β-D-Manp-(1→residues, as illustrated in Figure 4B [45,46,47,48].

A direct comparison of the NMR data highlights key structural differences between the two polysaccharides. FDOP presented seven anomeric proton signals, indicating a greater diversity of glycosyl residues in its backbone, which includes →4)-α-D-Manp-(1→ and →4)-β-D-Glcp-(1→ units not present in DDOP. In comparison, DDOP showed only six anomeric signals and a simpler backbone structure. It is suggested that traditional processing of fresh DO into Fengdou likely cleaves and rearranges polysaccharide chains, leading to the loss or reduction of specific α-D-Manp and β-D-Glcp segments and resulting in the simplified structure observed in DDOP.

### 3.9. Evaluation of Constipation-Relieving Activity

**Figure 5:**
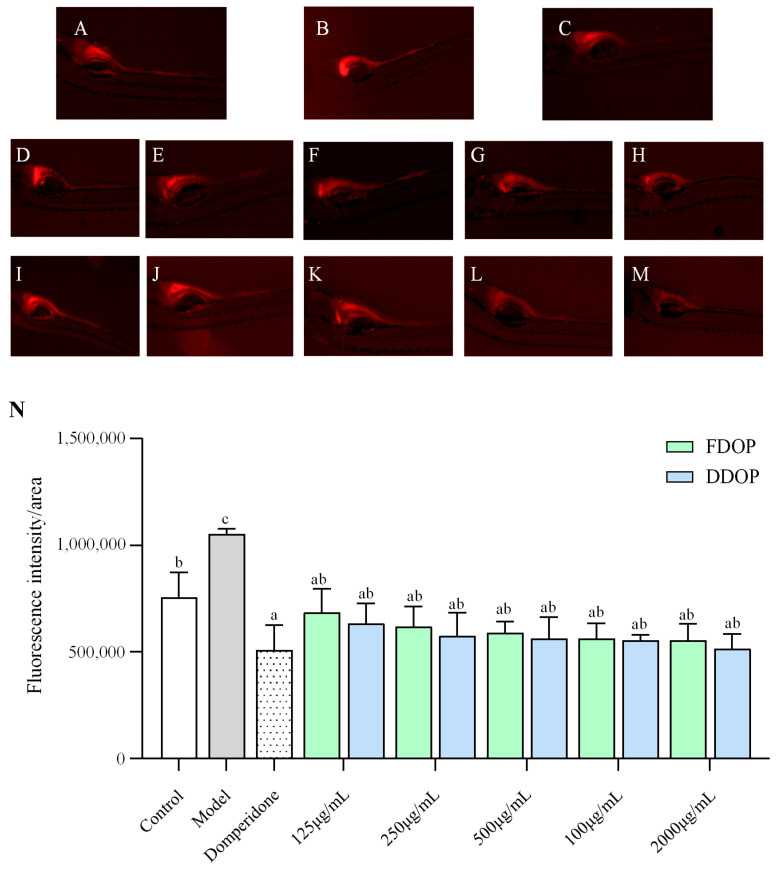
Results of relieving constipation activities in different groups of zebrafish. Control (**A**), Model (**B**), Domperidone (**C**), FDOP: 125, 250, 500, 1000, 2000 µg/mL (**D**–**H**), DDOP: 125, 250, 500, 1000, 2000 µg/mL (**I**–**M**), fluorescence intensity of FDOP and DDOP (**N**), White, gray, and dotted bars denote the control, model, and domperidone groups, respectively; colored bars denote FDOP and DDOP treatments. Data are presented as mean ± SD (*n* = 30 larvae per group). Statistical analysis was performed using one-way ANOVA followed by Tukey’s HSD post hoc test. Bars with different letters are significantly different (*p* < 0.05); bars sharing at least one letter are not significantly different.

The effects of FDOP and DDOP on the distribution of Nile Red in the zebrafish intestine are illustrated in Figure 5. Compared to the control group, the model group exhibited a significantly higher intestinal fluorescence intensity (*p* < 0.05), supporting the successful establishment of a constipation-like phenotype under this assay. In contrast, treatment with domperidone, FDOP, or DDOP markedly attenuated the elevated intestinal fluorescence caused by loperamide (*p* < 0.01). Across the tested concentrations, fluorescence intensity appeared to decrease numerically with increasing FDOP or DDOP dose. However, these between-dose differences did not reach statistical significance under the current experimental design and sample size. Critically, Tukey’s HSD post hoc test following one-way ANOVA confirmed that all treatment groups were significantly different from the model group after Tukey correction. No statistically significant differences were detected between FDOP and DDOP at matched doses (*p* > 0.05), indicating comparable efficacy within the current experimental conditions.

Our structural analysis revealed critical differences between the two polysaccharides. DDOP has a higher glucose content, a lower molecular weight, and a notably simplified backbone structure. These structural distinctions may correlate with their functional profiles. Based on the literature, it is hypothesized that the lower molecular weight and simpler structural features of DDOP might favor its fermentation by gut microbiota, a potential pathway for modulating intestinal function [49,50]. Likewise, its higher glucose content could influence interactions with digestive components such as bile acids [51,52,53]. It is critical to note that these specific mechanistic interpretations remain hypothetical, as they were not directly measured in this study. Future work employing targeted assays is needed for validation. Overall, our zebrafish data demonstrate that both polysaccharides alleviate constipation. Importantly, these results represent preclinical, screening-level evidence. The Nile Red readout in zebrafish larvae, while valuable for initial bioactivity assessment, does not directly translate to human constipation outcomes. Differences in gastrointestinal anatomy, neuromuscular regulation, microbial ecology, and intestinal transit physiology between zebrafish and humans, as well as the short exposure duration and larval developmental stage, limit direct extrapolation to human health. Consequently, confirmation in mammalian models and ultimately in human studies using clinically meaningful endpoints is required.

### 3.10. Evaluation of Hypolipidemic Activity

#### 3.10.1. In Vivo Hypolipidemic Activity Test

**Figure 6:**
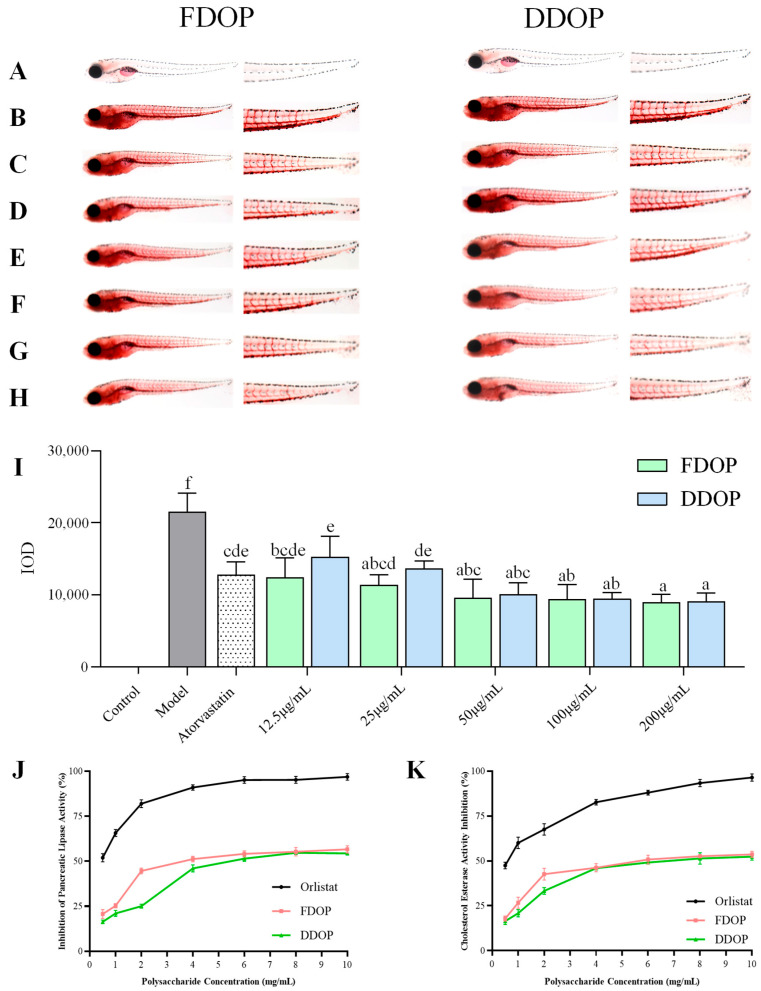
Results of hypolipidemic activities in different groups of zebrafish. Lipid accumulation levels in control (**A**), model (**B**), atorvastatin (**C**), 12.5, 25, 50, 100, 200 µg/mL of FDOP and DDOP (**D**–**H**), Integral optical density (IOD) of FDOP and DDOP (**I**), gray and dotted bars denote the model and atorvastatin groups, respectively; colored bars denote FDOP and DDOP treatments. Data in (**I**) are presented as mean ± SD (*n* = 30 larvae per group). Statistical analysis for (**I**) was performed using one-way ANOVA followed by Tukey’s HSD post hoc test. Bars with different letters are significantly different (*p* < 0.05). Bars sharing at least one letter are not significantly different. Pancreatic cholesterol esterase inhibition rate (**J**); Pancreatic lipase inhibition rate (**K**). Data in (**J**) and (**K**) are presented as mean ± SD (*n* = 3 independent replicates).

The hypolipidemic effects of FDOP and DDOP were evaluated in a zebrafish hyperlipidemia model using Oil Red O staining (Figure 6A–H). Larvae fed a high-fat diet containing egg yolk powder for 48 h exhibited marked lipid deposition in the caudal vasculature and abdominal region, whereas no staining was observed in the control group. The hyperlipidemic model was successfully established, as reflected by a significantly greater integrated optical density (IOD) in the model group compared to the control (*p* < 0.01). Treatment with atorvastatin, as well as FDOP or DDOP, reduced IOD values relative to the model group. Tukey’s HSD test following one-way ANOVA confirmed that all treated groups differed significantly from the model group (*p* < 0.05), indicating attenuated lipid deposition in zebrafish larvae. At the lower tested concentration (12.5 μg/mL, 25 μg/mL), FDOP showed numerically lower IOD values than DDOP. However, these differences between polysaccharides were observed as numerical trends rather than statistically significant effects after Tukey’s correction. As a screening tool, Oil Red O staining in zebrafish larvae provides rapid in vivo evidence of lipid modulation. Nevertheless, interspecies differences in lipid metabolism and physiology limit direct translation to human dyslipidemia. Accordingly, these findings should be viewed as preclinical evidence and require further validation in mammalian models.

#### 3.10.2. In Vitro Activity Assay

PCE, a key enzyme in lipid digestion with broad substrate specificity, hydrolyzes various lipids and is closely associated with the development of hyperlipidemia. Therefore, inhibiting enzyme activity is regarded as a potential strategy for preventing dietary cholesterol absorption [54]. As shown in Figure 6J,K, both FDOP and DDOP exhibited concentration-dependent inhibition of PCE and PL across a concentration span of 0.5–6.0 mg/mL. Beyond 6.0 mg/mL, no further increase in inhibition was observed, suggesting that the enzyme systems were approaching saturation. These findings corroborate earlier reports on the hypolipidemic activity of DO polysaccharides, which include inhibition of PCE and PL, reduction of lipid accumulation in zebrafish, and amelioration of dyslipidaemia in diabetic rats [55].

Under identical assay conditions, the differential efficacy of the two polysaccharides was further quantified by their half-maximal inhibitory concentration (IC_50_) values. FDOP exhibited lower IC_50_ values against both PCE (5.9 mg/mL) and PL (4.7 mg/mL) compared to DDOP (6.8 and 6.5 mg/mL, respectively), suggesting a stronger inhibition effect on these key digestive enzymes. Reported IC_50_ values for polysaccharide-based inhibition of PL or PCE vary across studies. For instance, a *Gracilaria lemaneiformis* polysaccharide fraction showed a PL IC50 of 2.923 ± 0.279 mg/mL [31], whereas polysaccharides from lingonberry press cake exhibited PL IC_50_ values of 5.33–6.24 mg/mL [56]. Such discrepancies are expected because the apparent IC_50_ is highly dependent on the experimental setup, including enzyme source, substrate system, pH, and ionic strength, incubation time, and correction for sample turbidity [57]. Therefore, the IC_50_ values obtained here should be interpreted primarily as a comparative index between FDOP and DDOP under identical assay conditions rather than as an absolute measure across different studies.

This superior activity is consistent with the distinct structural profile of FDOP, characterized by its higher molecular weight, greater mannose content, and more complex backbone architecture. These structural features could contribute to its bioactivity through several non-mutually exclusive, literature-based hypotheses. It has been suggested that higher-molecular-weight polysaccharides have a longer retention time in the intestinal tract and can bind with lipids such as cholesterol and excrete them from the body, thus lowering blood lipid levels [58]. Concurrently, the enriched mannose component may contribute specific bioactivity. Research by Wei et al. demonstrated that two mannose-rich polysaccharides isolated from Cordyceps militaris (IPCM-2 and EPCM-2, containing 51.94% and 44.51% mannose, respectively) significantly improved blood lipid profiles in hyperlipidaemic mice [59]. In addition, the more complex and extended backbone of FDOP may provide additional binding interfaces and stronger steric hindrance for binding to and inhibiting PL and PCE. Taken together, the more potent enzyme-inhibitory and overall hypolipidaemic activity of FDOP arises from a synergistic combination of its structural attributes, elevated molecular weight, mannose enrichment, and a structurally diversified backbone. Optimizing these parameters, particularly molecular weight and mannose-rich backbone domains, may therefore represent a promising strategy for enhancing the hypolipidaemic potential of DO-derived polysaccharides. However, these mechanistic explanations remain proposed interpretations and require further validation.

This study has several limitations that should be acknowledged. First, the zebrafish larval assays provide preclinical, screening-level evidence and rely on proxy endpoints (Nile Red fluorescence and Oil Red O/IOD), which do not directly translate to human outcomes. Second, the mechanistic interpretations proposed in the discussion (e.g., modulation of gut microbiota, SCFA production, bile acid sequestration) are hypotheses derived from correlating our structural/activity data with existing literature. They were not directly tested in the present study and require future experimental validation. Third, some observed bioactivity differences between FDOP and DDOP were numerical trends that did not reach statistical significance under our experimental conditions, warranting further investigation with adjusted paradigms or larger sample sizes. Addressing these limitations will be the focus of our future work.

## 4. Conclusions

In this study, polysaccharides were isolated from fresh *Dendrobium officinale* (FDOP) and its traditionally processed product, Fengdou (DDOP). Their structural characteristics were systematically compared, and their gastrointestinal and lipid-regulating activities were evaluated. Both polysaccharides were identified as mannose–glucose heteropolysaccharides. However, processing them induced significant structural changes. FDOP exhibited a higher molecular weight, a higher mannose-to-glucose ratio, and a more structurally diversified linked backbone, whereas DDOP showed a lower molecular weight, increased glucose proportion, and a simplified backbone architecture.

Functionally, both FDOP and DDOP significantly alleviated loperamide-induced constipation and reduced lipid accumulation in zebrafish models, demonstrating dual regulatory effects on intestinal motility and lipid metabolism. DDOP exerted a more pronounced constipation-relieving effect, which is likely related to its lower molecular weight and simplified backbone that may favor solubility, microbial fermentation, and bile acid interaction in the gut. On the other hand, FDOP displayed stronger inhibitory activity against pancreatic cholesterol esterase and pancreatic lipase, as well as superior lipid-lowering efficacy in vivo. These effects are attributable to its higher molecular weight, enriched mannose content, and more complex →4-linked backbone, which collectively provide greater steric hindrance and binding capacity toward lipid-digesting enzymes.

Overall, these findings indicate that traditional processing of DO not only improves stability and storability, but also reshapes the structure–activity profile of its polysaccharides. This differentiates FDOP and DDOP into functionally distinct yet complementary candidates for gut-metabolic modulation. The clarified relationships between polysaccharide structure and biological activity provide a useful rationale for the targeted design and optimization of DO-derived polysaccharides as natural ingredients for functional foods or adjuvant interventions aimed at constipation and dyslipidaemia.

## Figures and Tables

**Table 1:** Linkage analysis of FDOP and DDOP by methylation and GC-MS.

No.	Methylation Debris	Major Fragments (*m*/*z*)	Types of Linkages	Molecular Ratio %	
				FDOP	DDOP
1	1,5-di-*O*-acetyl-(1-deuterio)-2,3,4,6-tetra-*O*-methyl glucitol	59.0, 71.7, 87.0, 102.0, 111.8, 129.0, 145.1, 161.0, 205.1	*t*-Glcp	11.1	20.9
2	1,5-tri-*O*-acetyl-(1-deuterio)-2,3,6-tri-*O*-methyl-4-hydroxyl-manitol	59.0, 75.0, 88.0, 102.0, 118.0, 131.0, 162.0, 191.1	1,4-Manp(3-*O*-Ac)	15.9	30.3
3	1,5-tri-*O*-acetyl-(1-deuterio)-2,3,6-tri-*O*-methyl-4-hydroxyl-glcitol	59.0, 75.0, 88.0, 102.0, 118.0, 131.0, 162.0, 191.1	1,4-Glcp(3-*O*-Ac)	7.9	11.7
4	1,4,5-tri-*O*-acetyl-(1-deuterio)-2,3,6-tri-*O*-methyl-manitol	99.0, 118.0, 142.0, 162.0, 233.1	1,4-Manp	57.1	30.4
5	1,4,5-tri-*O*-acetyl-(1-deuterio)-2,3,6-tri-*O*-methyl-glucitol	99.0, 118.0, 142.0, 162.0, 233.1	1,4-Glcp	8.1	6.7

## Data Availability

The original contributions presented in the study are included in the article/Supplementary Material; further inquiries can be directed to the corresponding authors.

## Supplementary Materials

The following supporting information can be downloaded at: https://www.mdpi.com/article/10.3390/foods15040727/s1, Figure S1: ^1^HNMR (A) and ^13^C NMR (B) of FDOP; Figure S2: ^1^HNMR (A) and ^13^C NMR (B) of DDOP; Table S1: Mobile phase gradient elution conditions; Table S2: The ^1^H (700 MHz) and ^13^C (175 MHz) NMR data of FDOP; Table S3: The ^1^H (700 MHz) and ^13^C (175 MHz) NMR data of DDOP.
