# Supplementary material for: Structural Characterization, Constipation-Relieving, and Hypolipidemic Activity of Polysaccharides from Fresh and Processed Dendrobium officinale
Source: Foods. 2026 Feb 15;15(4):727. doi: 10.3390/foods15040727 (PMC12939700; doi:10.3390/foods15040727)
Supplement: Supplementary file 1 [file foods-15-00727-s001.zip › foods-4102204-SI.pdf]

No.	Content
**Figure S1**	^{1}HNMR (A) and ^{13}C NMR (B) of FDOP.
**Figure S2**	^{1}HNMR (A) and ^{13}C NMR (B) of DDOP.
**Table S1**	Mobile phase gradient elution conditions.
**Table S2**	The ^{1}H (700 MHz) and ^{13}C (175 MHz) NMR data of FDOP
**Table S3**	The ^{1}H (700 MHz) and ^{13}C (175 MHz) NMR data of DDOP

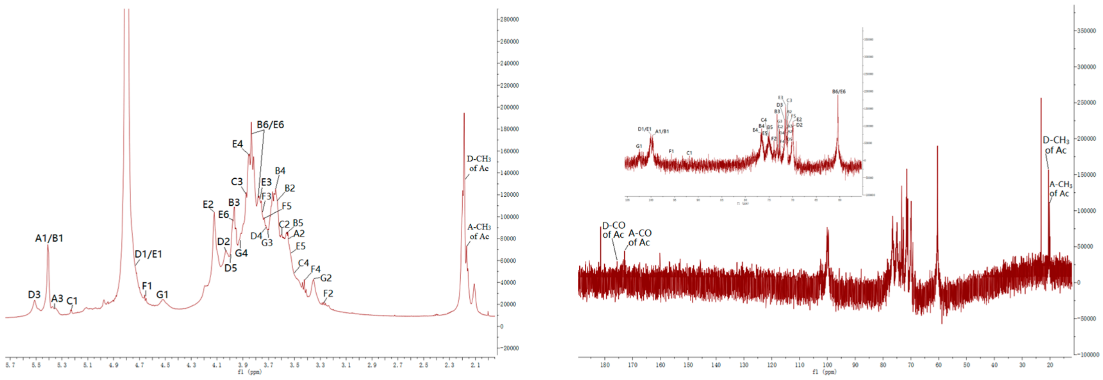

（A） （B）

Figure S1 ^{1}HNMR (A) and ^{13}C NMR (B) of FDOP.

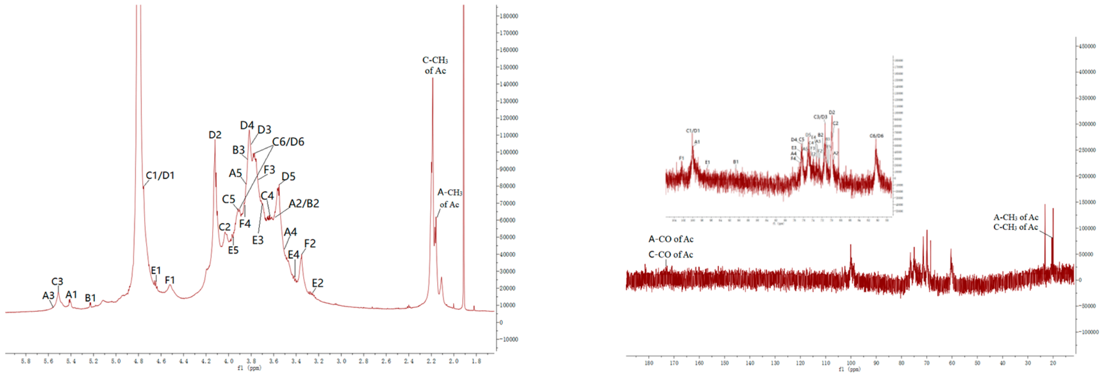

（A）　　　　　　　　　　　　　（B）

Figure S2 ^{1}HNMR (A) and ^{13}C NMR (B) of DDOP.

Table S1 Mobile phase gradient elution conditions

Time（min）	0	20	20.1	35	35.1	45	45.1	55
A(H_2O)/%	94	94	89	74	20	20	94	94
B(250 mM NaOH)/%	6	6	6	6	80	80	6	6
C(1 M NaAC)/%	0	0	5	20	0	0	0	0

Note: The “-” indicates not detected.

Table S2 The ^{1}H (700 MHz) and ^{13}C (175 MHz) NMR data of FDOP

			1	2	3	4	5	6	CH_3 of Ac	CO of Ac
A	→4)-3-O-acetyl-α-D-Glcp-(1→	δ_{H}	5.39	3.52	5.35	-	-	-	2.16	-
		δ_{C}	99.71	71.21	71.42	-	-	-	20.26	173.88
B	→4)-α-D-Manp-(1→	δ_{H}	5.39	3.62	3.96	3.65	3.59	3.76,3.85	-	-
		δ_{C}	99.71	71.49	73.84	76.57	74.98	60.40	-	-
C	t-α-D-Glcp(1→	δ_{H}	5.22	3.58	3.88	3.51	-	-	-	-
		δ_{C}	91.72	71.59	71.00	74.80	-	-	-	-
D	→4)-3-O-acetyl-β-D-Manp-(1→	δ_{H}	4.73	4.01	5.50	3.70	3.96	-	2.19	-
		δ_{C}	100.08	69.95	71.32	72.80	71.18	-	20.51	173.10
E	→4)-β-D-Manp-(1→	δ_{H}	4.73	4.11	3.63	3.84	3.53	3.97	-	-
		δ_{C}	100.08	69.93	71.49	76.61	75.25	60.40	-	-
F	t-β-D-Glcp(1→	δ_{H}	4.65	3.27	3.76	3.41	3.73	-	-	-
		δ_{C}	96.95	73.84	75.97	72.56	71.53	-	-	-
G	→4)-β-D-Glcp-(1→	δ_{H}	4.51	3.34	3.72	-	-	-	-	-
		δ_{C}	102.44	72.82	72.99		-	-	-	-

Note: The “-” indicates not detected.

Table S3 The ^1H (700 MHz) and ^{13}C (175 MHz) NMR data of DDOP

			1	2	3	4	5	6	CH_3 of Ac	CO of Ac
A	→4)-3-*O*-acetyl-α-D-Glc*p*-(1→	δ_H	5.41	3.59	5.57	3.51	3.80	-	2.15	-
		δ_C	99.29	69.47	72.77	76.44	74.80	-	20.14	173.64
B	*t*-α-D-Glc*p*(1→	δ_H	5.23	3.57	3.81	-	-	-	-	-
		δ_C	91.73	71.49	71.03	-	-	-	-	-
C	→4)-3-*O*-acetyl-β-D-Man*p*-(1→	δ_H	4.76	4.03	5.52	3.61	3.91	3.75	2.19	-
		δ_C	100.05	69.85	71.31	74.80	76.31	60.40	20.14	173.35
D	→4)-β-D-Man*p*-(1→	δ_H	4.76	4.12	3.82	3.81	3.55	3.75/3.92	-	-
		δ_C	100.05	69.92	71.37	76.42	74.97	60.40	-	-
E	*t*-β-D-Glc*p*(1→	δ_H	4.65	3.26	3.68	3.34	3.99	-	-	-
		δ_C	95.59	73.78	76.47	72.57	71.09	-	-	-
F	→4)-β-D-Glc*p*-(1→	δ_H	4.53	3.31	3.75	3.87	-	-	-	-
		δ_C	102.42	72.71	74.82	76.30	-	-	-	-

Note: The "-" indicates not detected.
